# Characterization of Cells Isolated from Genetic and Trauma-Induced Heterotopic Ossification

**DOI:** 10.1371/journal.pone.0156253

**Published:** 2016-08-05

**Authors:** Shailesh Agarwal, James Drake, Ammar T. Qureshi, Shawn Loder, Shuli Li, Kay Shigemori, Jonathan Peterson, David Cholok, Jonathan A. Forsberg, Yuji Mishina, Thomas A. Davis, Benjamin Levi

**Affiliations:** 1 Department of Surgery, University of Michigan Health Systems, Ann Arbor, MI, 48109, United States of America; 2 Department of Regenerative Medicine, Naval Medical Research Center, Silver Spring, MD, 20910, United States of America; Second University of Naples, ITALY

## Abstract

Heterotopic ossification (HO) is the pathologic formation of bone separate from the normal skeleton. Although several models exist for studying HO, an understanding of the common *in vitro* properties of cells isolated from these models is lacking. We studied three separate animal models of HO including two models of trauma-induced HO and one model of genetic HO, and human HO specimens, to characterize the properties of cells derived from tissue containing pre-and mature ectopic bone in relation to analogous mesenchymal cell populations or osteoblasts obtained from normal muscle tissue. We found that when cultured *in vitro*, cells isolated from the trauma sites in two distinct models exhibited increased osteogenic differentiation when compared to cells isolated from uninjured controls. Furthermore, osteoblasts isolated from heterotopic bone in a genetic model of HO also exhibited increased osteogenic differentiation when compared with normal osteoblasts. Finally, osteoblasts derived from mature heterotopic bone obtained from human patients exhibited increased osteogenic differentiation when compared with normal bone from the same patients. These findings demonstrate that across models, cells derived from tissues forming heterotopic ossification exhibit increased osteogenic differentiation when compared with either normal tissues or osteoblasts. These cell types can be used in the future for *in vitro* investigations for drug screening purposes.

## Introduction

Heterotopic ossification (HO), the pathologic formation of ectopic bone in soft tissues, occurs after extremity trauma, burn injury, spinal cord, traumatic brain injuries and orthopaedic procedures.[1–7] Additionally, a point mutation in *ACVR1* causes fibrodysplasia ossificans progressiva (FOP), a debilitating condition in which heterotopic bone forms independent of trauma.[8] Due to the variety of settings in which HO occurs, several models have been developed to examine *in vivo* treatment strategies including mouse and rat trauma models, and transgenic mouse models[9–15]. Critical to the interrogation of these *in vivo* models is the development of representative *in vitro* cell studies which can be used as a screening tool, especially in the context of studying possible pharmacologic targets. One major barrier to creating representative *in vitro* studies however, has been the challenge of identifying the tissue origin of the progenitor cell populations which form HO under a variety of trauma-induced conditions. Some studies have utilized muscle resident stromal cells or adipose-derived mesenchymal cells to evaluate *in vitro* osteogenic differentiation potential[16–18]. Other studies have utilized cells from patients with fibrodysplasia ossificans progressiva (FOP), although these may not be readily available[19]. However, these cell populations do not represent the heterogeneous cell population that is present in foci of developing HO lesions. It is critical to establish representative cellular assays which may be used to rapidly study treatment strategies. In this study, we examine four separate models of heterotopic ossification including two murine trauma models[16, 17, 20–23], a transgenic murine model of genetic HO[24], and human HO to demonstrate the *in vitro* characteristics of tissues which have either developed HO or will become HO. These models have been selected to represent the clinical scenarios during which heterotopic ossification forms—in situations of high-energy trauma as with our two murine trauma models, and in situations of genetic mutation. The use of human HO samples is intended to represent a more clinically translatable sample, and are derived from patients who developed HO subsequent to trauma. Our characterization of these cell lines will validate further *in vitro* analyses of potential therapeutics using these cells.

## Materials and Methods

### Animals

C57BL/6 male mice from Charles River (Wilmington, MA) were used for all experiments with old (16–18 months old) mice between and young (6–8 weeks old) mice. Adult male Sprague Dawley rats (450-600g) were obtained from Taconic Farms (Germantown, NY). All animals were housed in clean plastic cages and kept on a 12-hour light/dark cycle with unlimited access to food (standard rodent chow) and fresh water ad libitum. Animals were acclimated for at least two weeks prior to experimentation. All animal procedures were carried out in accordance with the guidelines provided in the Guide for the Use and Care of Laboratory Animals from the Institute for Laboratory Animal Research (ILAR, 2011). The study protocols were reviewed and approved by the Institutional Animal Care and Use Committee of the University of Michigan (PRO0001553) and were reviewed and approved by the Walter Reed Army Institute of Research/Naval Medical Research Center Institutional Animal Care and Use Committee (12-OUMD-20S) in compliance with all applicable Federal regulations governing the protection of animals in research. In our genetic model, we crossed mice carrying the conditional constitutively active allele of *ACVR1* (*ACVR1* carrying Q207D mutation, *ca-ACVR1*) with *Nfatc1-Cre* transgenic mice. F1 offspring mice carrying both transgenes (Nfatc1-cre^+^/caACVR1^fx/wt^) were used as experimentals whereas Cre^-^ or Nfatc1-cre^+^/caACVR1^wt/wt^ littermates were used as controls.[25–29]

### Burn/tenotomy injury

All mice used for analysis received a partial thickness burn injury as previously described^23^. Briefly, animals were anesthetized with 3–5% inhaled isoflurane. Hair was closely clipped on the left dorsum to expose the skin. Partial-thickness burn was achieved by placing a metal brand, heated to 60°C in a water bath, against the exposed skin for 18 seconds. Each mouse then received a concurrent Achilles tenotomy with sharp dissection at the midpoint in the left leg. Pain management was achieved with subcutaneous injections of buprenorphine every 12 hours for 3 days. Mice were euthanized using carbon dioxide and cervical dislocation. At indicated time points post injury, tissue surrounding the tendon transection site was collected in addition to tendon from the same region on the uninjured contralateral hindlimb. Mouse mesenchymal cells (mMSCs) were digested and cultured as previously described[30].

### Extremity Polytrauma and Hind Limb Amputation Model

Rats were anesthetized with isoflurane and received buprenorphine (0.05mg/kg) delivered via intraperitoneal injection and were exposed whole body blast overpressure (120 ± 7 kPa) via a pneumatically driven shock tube. A drop weight apparatus (University of Alabama, Birmingham, AL) was used to create a comminuted femur fracture in a similar fashion to that previously described[31]. The weight was dropped from a height of 88 cm, which reliably reproduced a comminuted mid-shaft femur fracture. Immediately after, a soft-tissue crush injury was created by rotating the fracture site between the two anvils of the support stage. Twenty pounds per square inch of pressure was applied for a period of one minute as determined using a Chatillon force measurement pressure sensor (AMETEK Inc., Largo, FL). Immediately following the femur fracture and crush injury, we placed a circumferential incision around the distal femur releasing the extensor mechanism and hamstrings distally. The amputation was made through the fracture, in an effort to simulate an amputation performed within the zone of injury. This is a common practice when treating blast wounds, in which the zone of injury is extensive. Devitalized bone was debrided and hemostasis achieved. Next, we performed a myoplasty by suturing the distal hamstring and quadriceps fascia over the residual femur, and closed the amputation wound with 3–0 polyglactin 910 and 4–0 poliglecaprone 25 suture. Post operatively, all animals received sustained release buprenorphine (1.2 mg/kg SC; Zoopharm, Windsor, CO) with repeat dosing after three days. Animals were monitored twice a day for pain management and wound healing[20–22].

At indicated time points post injury, rats were euthanized with pentobarbital (Fatal Plus; 390 mg/kg intraperitoneally; Patterson Veterinary, Devens, MA, USA). Tissue surrounding the amputation site was collected from the residual limb in addition to muscle tissue from the same region on the uninjured contralateral hind limb. Rat mesenchymal cells (rMSCs) were digested and cultured as previously described[30].

### Human HO tissue Harvest

All human subjects and animal research approval was provided by the University of Michigan and Naval Medical Research Center. All participants provided written informed consent to participate in this study. Written consent was approved by the IRB at the University of Michigan. All patients were between the ages of 20 and 38 and none of them had other significant co-morbidities or were on medications related to osteoblast biology. Ectopic heterotopic bone and normal surrounding skeletal bone were excised using normal surgical technique without excising excess tissue. The ectopic bone and skeletal bone tissues were immediately separated and placed in sterile saline. Some of the tissue was processed for histology. The remaining tissues were then vigorously mechanically disrupted with a rongeur and then placed into standard growth medium. Cells were allowed to migrate out of the bone fragments and then subsequently expanded in culture dishes containing Dulbecco’s Modified Eagle Medium (DMEM; ThermoFisher Scientific) (vendor, City, State), 10% FBS, 1% Pen/Strep, 2mM L-Glut, and used at passages 1–4. All *in vitro* culture experiments were run using actively proliferating cells harvested (trypsin-EDTA treatment) from semi-confluent monolayer of adherent cells derived from ectopic heterotopic bone and non-heterotopic skeletal bone tissue.

### In vitro osteogenic differentiation of cells

Cells (passage 1–4) were resuspended in osteogenic differentiation medium (ODM), seeded in triplicate onto six-well plates at a density of 1× 10^5^ cells/well and onto a 12-well plate at a density of 3.5 × 10^4^ cells/well, and then cultured at 37°C in 5% CO_2_ in air as previously described.[32] ODM was changed every 3 days. Early osteogenic differentiation was assessed by alkaline phosphatase (ALP) stain and quantification of ALP enzymatic activity after 7 days as previously described[32]. Alizarin red staining for bone mineral deposition and colorimetric quantification was completed at 2 weeks as previously described [32].

### Quantitative RT-PCR

RNA was harvested from cells after 7 days in ODM using RNeasy Mini Kit (Qiagen, Germantown, MD) according to manufacturer’s specifications. Reverse transcription was performed with 1 μg RNA using Taqman Reverse Transcription Reagents (Applied Biosystems, Foster City, CA). Quantitative real-time PCR was carried out using the Applied Biosystems Prism 7900HT Sequence Detection System and Sybr Green PCR Master Mix (Applied Biosystems) as previously described[32]. Specific primers for these genes were chosen based on their PrimerBank sequences (S1 Table).

### Western blot analysis

Cultured cells were lysed and protein collected after 7 days differentiation in ODM, separated on polyacrylamide gels, transferred to polyvinylidene fluoride membranes, and assayed with standard immunoblotting technique using the following primary antibodies: anti-phospho-Smad 1/5/8, anti-Smad 5, (Cell Signaling Technologies, Boston, MA) as previously described.[33]

### Cell proliferation assays

Tissue derived cells (P1-4) were seeded in 12-well plates at a density of 5× 10^3^ cells per well (*n* = 3). Cells were grown in standard growth medium. At days 1, 3, 5, and 7 the numbers of viable cells harvested cells following trypsin-EDTA treatment were manually enumerated using Trypan blue stain and a hemocytometer. Additionally, cell proliferation was assessed by bromodeoxyuridine (BrdU) incorporation as previously described[34].

### FACS analysis

Tissue was isolated from the injured mouse specimens, or alternatively cells were isolated from the culture plate. FACS analysis was then performed to interrogate specific subpopulations of cells present in the isolated tissue or from the isolated cells. All collected tissues were mechanically dissociated using sterile scissors. Tissues were digested for 120 minutes in 0.75% Collagenase 2 (Sigma-Aldrich) in Hanks Balanced Salt Solution (HBSS) at 37C under sustained agitation. Samples were filtered using successive 100- and 70-micron sterile strainers and digestion was quenched using equal parts 10% DMEM. Samples were centrifuges at 1,000 rpm for 5 minutes before removing the supernatant and washing in HBSS. This process was repeated in triplicate.

Staining for osteoproliferative cells was performed using previously described markers for osteogenic and proliferative stromal cells in musculoskeletal injury[35, 36]. Antibodies used included: CD11b-efluor450 (48-0112-82; Affymetrix (eBioscience)), CD34-FITC (11-0341-82; Affymetrix (eBioscience)), CD31-APC (17-0311-82; Affymetrix (eBioscience)), CD45-PECyanine7 (25-0451-82; Affymetrix (eBioscience)), and Anti-Integrin beta 1 Antibody (Abcam). Samples were incubated for 1 hour at 4°C then washed and stained an additional 30 minutes with Donkey Anti-Mouse IgG H&L (Alexa Fluor^®^ 594). Following incubation, samples were washed three times and filtered through a 45 micron mesh before being run on a FACSAria II (BD Biosciences) Cell Sorter at the University of Michigan Flow Cytometry Core. Greater than 10,000 events were collected for each population. All analyses were carried out in FlowJo.

### Statistical analysis

Data were analyzed using SPSS software (v21, IBM). Means and standard deviations were calculated from numerical data and statistical analysis was performed using an appropriate ANOVA. Equivariance was assessed with Levene’s test and a Welch correction was applied when indicated. Post-hoc analysis was completed with Tukey’s test with equivariant data or Games-Howell if Levene’s test failed. In the figures, the bar graphs represent mean of independent experimental replicates, and error bars represent one standard deviation. For all analyses, significance was defined as a p < 0.05.

## Results

### HO-derived osteoblasts are more proliferative than non-HO osteoblasts

**Fig 1:**
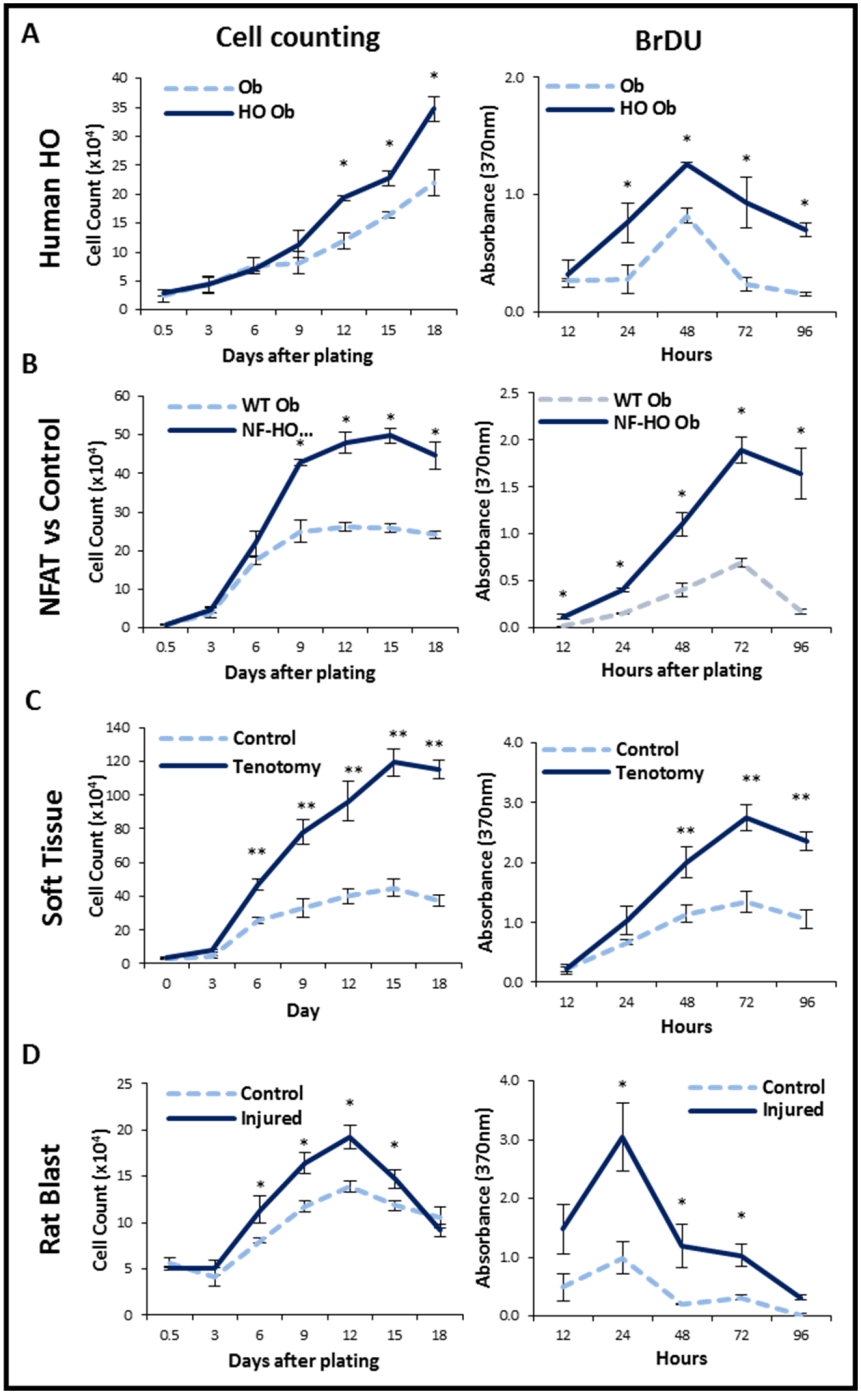
Characterization of osteoblasts and MSC *in vitro* cell proliferation using cells isolated and expanded from 4 sources of HO tissue. (A) Human osteoblasts cultured from heterotopic bone (HO-Ob) demonstrated higher proliferation rates by cell counting (left) and BrDU (right) than osteoblasts derived from normal bone (Ob). (B) Osteoblasts from a genetic model of HO (NF-HO) in which the BMP receptor demonstrated higher proliferation rates compared to osteoblasts from WT mice (WT Ob) by cell counting (left) and BrDU (right). (C) In a mouse tenotomy/burn model of HO, mesenchymal cells derived from soft tissue harvested from the tenotomy site (Tenotomy) demonstrated higher proliferation than cells taken from soft tissue from the contralateral, non-tenotomized leg (Control) by cell counting (left) and BrDU (right). (D) In a blast/polytraumatic extremity injury model in rats, osteoblasts from the injured side (Injured) were more proliferative than cells from the non-injured side (control). Cell proliferation was determined by manual hemocytometer cell counts using Trypan blue stain (left) and BrDU incorporation (right). Data are means ± SD. *p<0.05; **p<0.01. Student T-test.

We first compared the proliferative capacity of osteoblasts derived from HO lesions with that of osteoblasts from normal skeletal osteoblasts using transgenic mice (*Nfatc1-cre/caACVR1*^*fl/wt*^) and human patients. Based on BrDU labeling and manual cell counts, we noted that HO-derived osteoblasts in both settings were significantly more proliferative than their respective normal skeletal osteoblasts (Fig 1).

### mMSCs and rMSCs derived from the trauma site are more proliferative than cells derived from the uninjured site

Because our transgenic model and human HO samples do not allow for examination of tissues in the setting of recent trauma, we next evaluated two separate models of trauma-induced HO. Tissue was harvested from the tendon transection site or uninjured hind limb of burn/tenotomy mice (mMSCs), and from the amputation site or contralateral hind limb of rats following blast and polytraumatic extremity injury (rMSCs). Cells derived from the soft tissue of the tenotomy site were significantly more proliferative when compared with those from the contralateral hind limb based on BrDU and manual cell counts (Fig 1). Similarly, soft tissue from the injury site of rats exposed to blast and polytramautic extremity injury also exhibited increased proliferation when compared with cells derived from the contralateral hind limb which was exposed to only blast overpressure exposure (Fig 1). Thus, mMSCs or rMSCs whether derived from a tendon injury or muscle injury both remain more proliferative than their uninjured controls.

### Cells derived from pre- and mature HO tissue exhibit increased osteogenic differentiation

**Fig 2:**
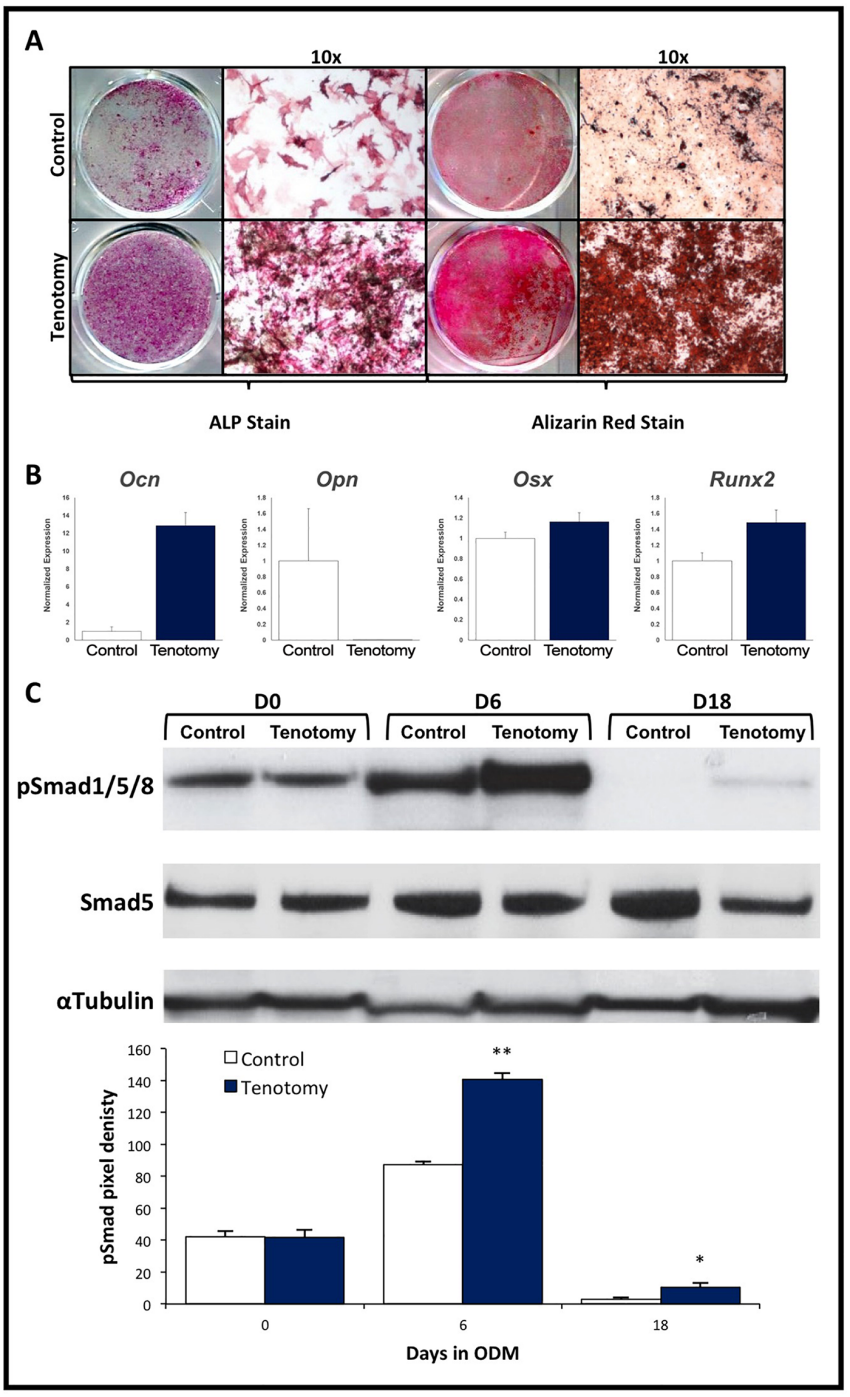
Characterization of *in vitro* osteogenic differentiation of muscle-derived MSCs isolated from a mouse tenotomy/burn model of HO. (A) MSCs were harvested and cultured from the soft tissue about the ankle in a mouse tenotomy/burn model that demonstrates ectopic bone development 4–6 weeks after injury. MSCs harvested from the tenotomy site (Tenotomy) 5 days after injury showed more ALP activity and more in-vitro bone deposition by Alizarin red staining than cells derived from the same site on the contralateral, un-injured leg (Control). Representative wells and 10x micrographs are shown. (B) Gene transcript levels of *Ocn*, *Opn*, *Osx*, and *Runx2*. (C) Western blot analysis of pSmad 1/5 protein levels in these cells after 0, 6, and 18 days in osteogenic media. Data shown are reported as the mean ± SD of at least three separate experimental samples performed in triplicate. *p<0.05; **p<0.01. Student T-test.

**Fig 3:**
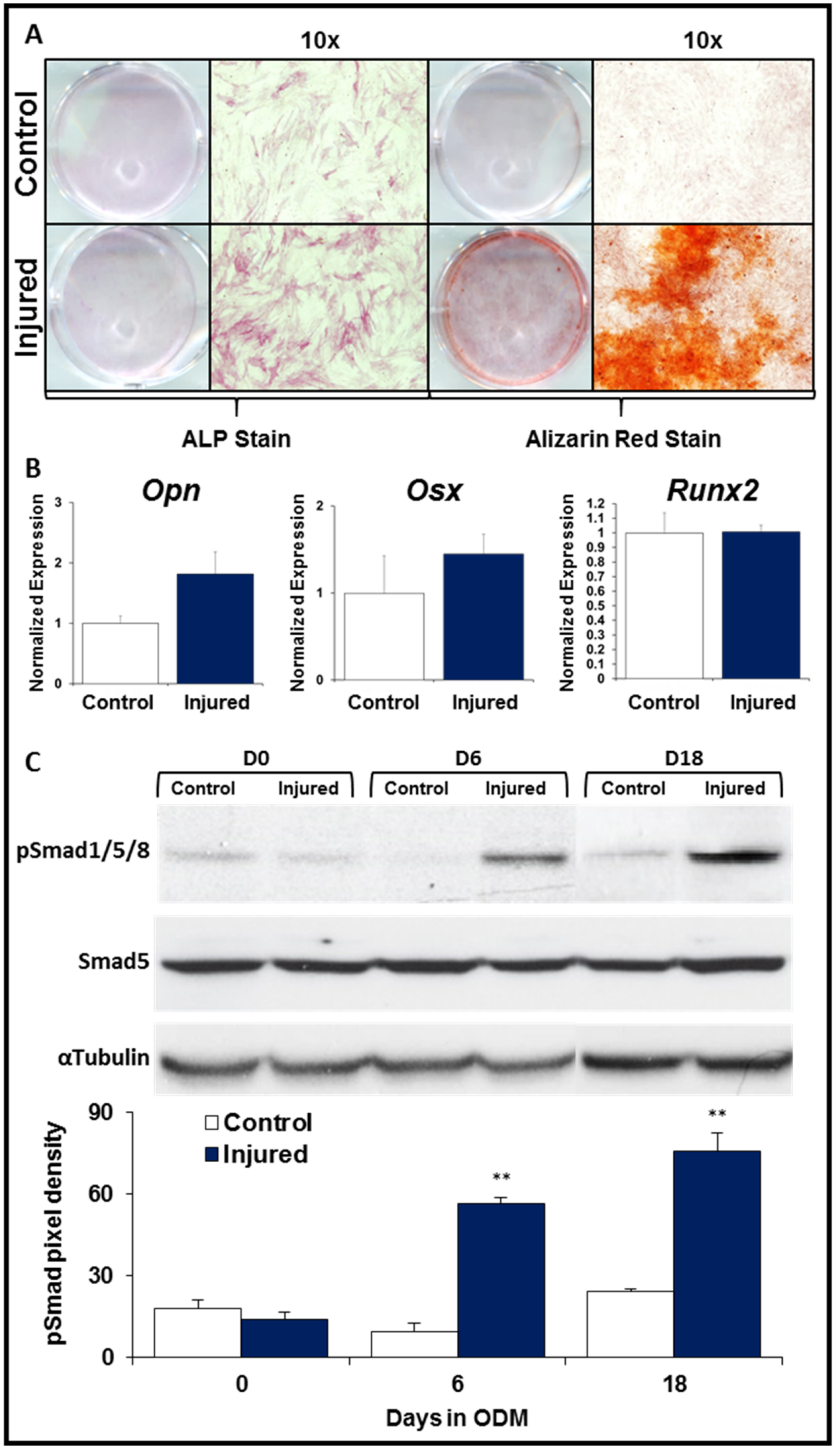
Characterization of *in vitro* osteogenic differentiation of muscle-derived MSC isolated from a rat blast/polytraumatic extremity injury model of HO. (A) MSCs were harvested and cultured from the soft tissue about the injury site in a rat blast/ polytraumatic extremity injury model. MSCs harvested from the blast injury/amputation site (Injured) 5 days after injury showed more ALP activity and more in vitro bone deposition by Alizarin red staining than cells derived from the same site on the contralateral, un-injured limb (Control). Representative wells and 10x micrographs are shown. (B) Expression levels of *Opn*, *Osx*, and *Runx2*. (C) Western blot analysis of pSmad 1/5 protein levels in these cells after 0, 6, and 18 days in osteogenic media. Data shown are reported as the mean ± SD of at least three separate experimental samples performed in triplicate. *p<0.05; **p<0.01. Student T-test.

We next established whether cells derived from pre-HO and mature HO tissue had a greater propensity *in vitro* to differentiate into osteoblasts and produce mineralized matrix. After culture in osteogenic differentiation medium (ODM), mMSCs from the tenotomy site of the burn/tenotomy model had significantly more alkaline phosphatase expression and mineral deposition (alizarin red) when compared with cells derived from the contralateral uninjured hind limb (Fig 2). Further gene expression analysis confirmed these findings, demonstrating elevated expression of pro-osteogenic genes including *Runx2* and *Ocn* (Fig 2). Finally, expression of the down-stream regulator of osteogenic gene expression, phospho-Smad 1/5/8 was also up-regulated in cells derived from the traumatic soft tissue (Fig 2). These results were confirmed using rMSCs isolated from wounds in the rat blast/polytraumatic extremity injury model (Fig 3), indicating that the trauma site in this model also exhibits increased osteogenic differentiation. These findings confirm that cells from sites forming HO exhibit increased osteogenic potential.

### Cells derived from pre-HO tissue are enriched with osteoproliferative cells

**Fig 4:**
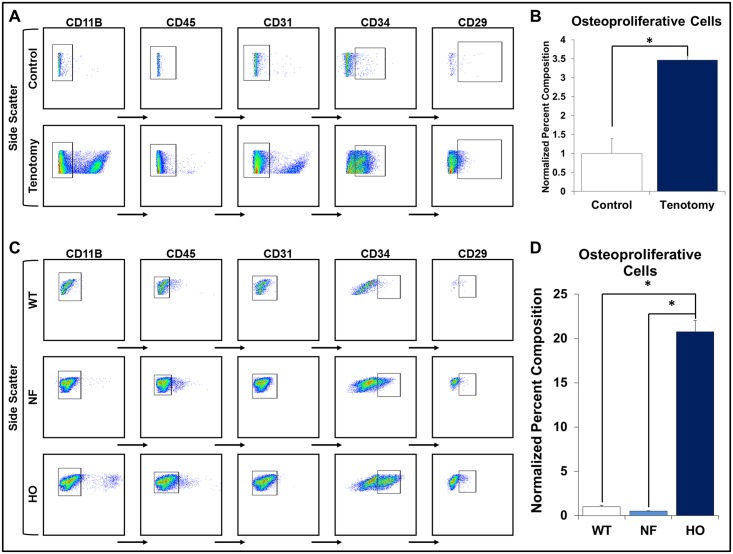
FACS analysis of early post-injury tissue and HO osteoblasts. (A) FACS gating for osteoproliferative cells from tendon transection site one week after injury and contralateral hindlimb; (B) Bar graph showing quantification; (C) FACS gating for osteoproliferative cells from HO and normal osteoblasts of mutant mice and wild type osteoblasts; (D) Bar graph showing quantification. All experiments performed in triplicate. * = p<0.05.

Tissue was isolated from the tendon transection site after one week and analyzed using FACS. When compared with the uninjured contralateral hindlimb, injury-site tissue was enriched 3.5-fold (p<0.05) for CD34+CD29+CD31-CD45-CD11b-[35, 36] osteoproliferative cells (Fig 4). Additionally, HO-derived osteoblasts from mutant mice (*Nfatc1-cre/caACVR1*^*fl/fl*^) had a 20.8-fold enrichment (p<0.05) of these osteoproliferative cells when compared with wild type osteoblasts and 43.0-fold enrichment (p<0.05) when compared with normal femur osteoblasts isolated from mutant mice (Fig 4).

### HO-derived osteoblasts exhibit increased osteogenic differentiation when compared with skeletal osteoblasts

**Fig 5:**
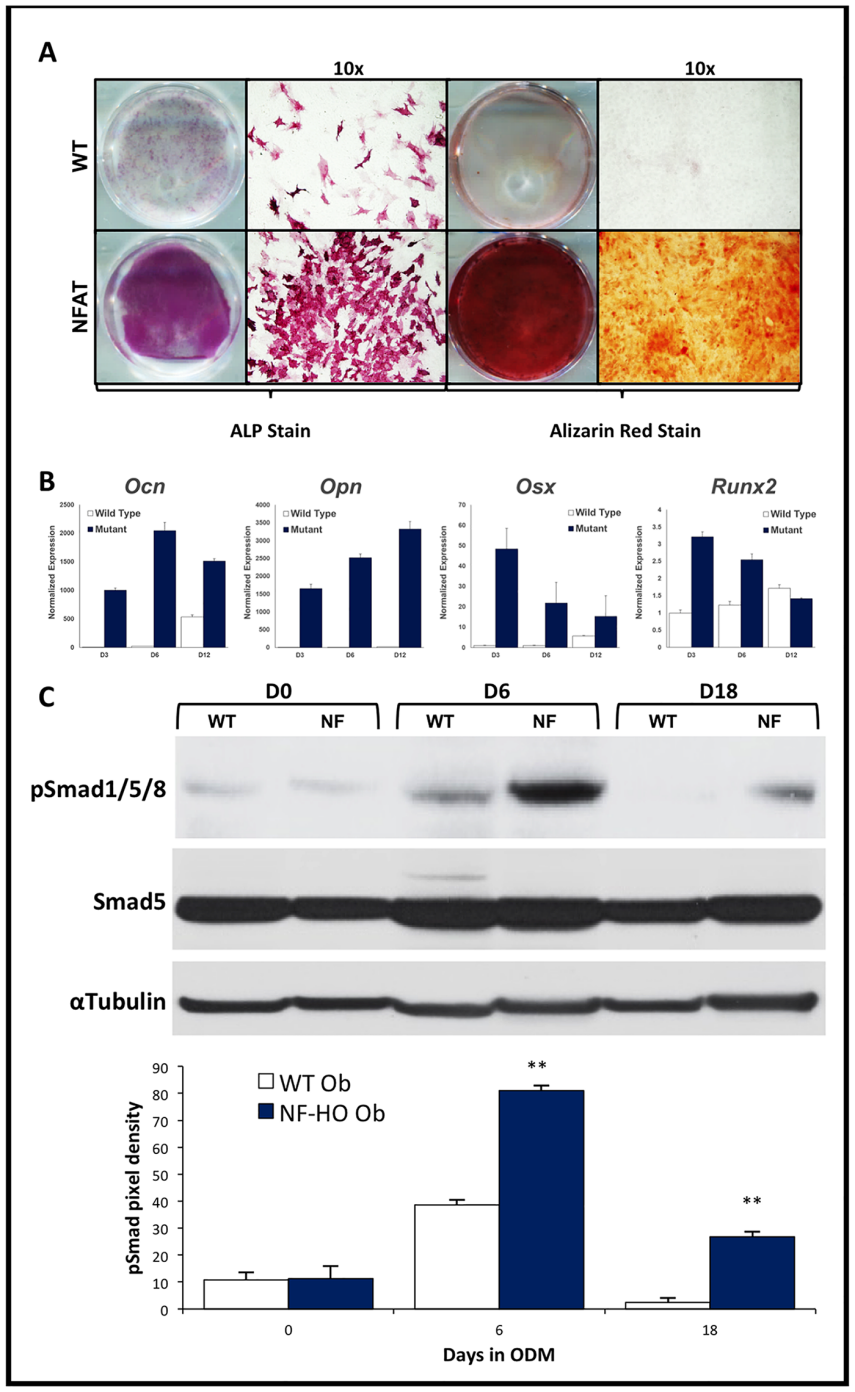
Characterization of *in vitro* osteogenic differentiation of osteoblasts isolated from a genetic model of HO in mice. (A) Osteoblasts were harvested and cultured from ectopic bone produced in mice with a constitutively active form of the BMP receptor ALK2 in cells expressing NFAT and from normal bone of WT mice. Cells harvested from the mutant mice (NF-HO) showed more ALP activity and more in-vitro bone deposition by Alizarin red staining than cells derived from WT mice (WT Ob). Representative wells and 10x micrographs are shown. (B) Similarly, these cells from the mice with constitutively active ACVR1 demonstrated increased expression of osteogenic gene transcripts for *Ocn*, *Opn*, *Osx*, and *Runx2*. (C) Western blot analysis of pSmad 5 protein levels in these cells after 0, 6, and 18 days in osteogenic media. Data shown are reported as the mean ± SD of at least three separate experimental samples performed in triplicate. *p<0.05; **p<0.01. Student T-test.

**Fig 6:**
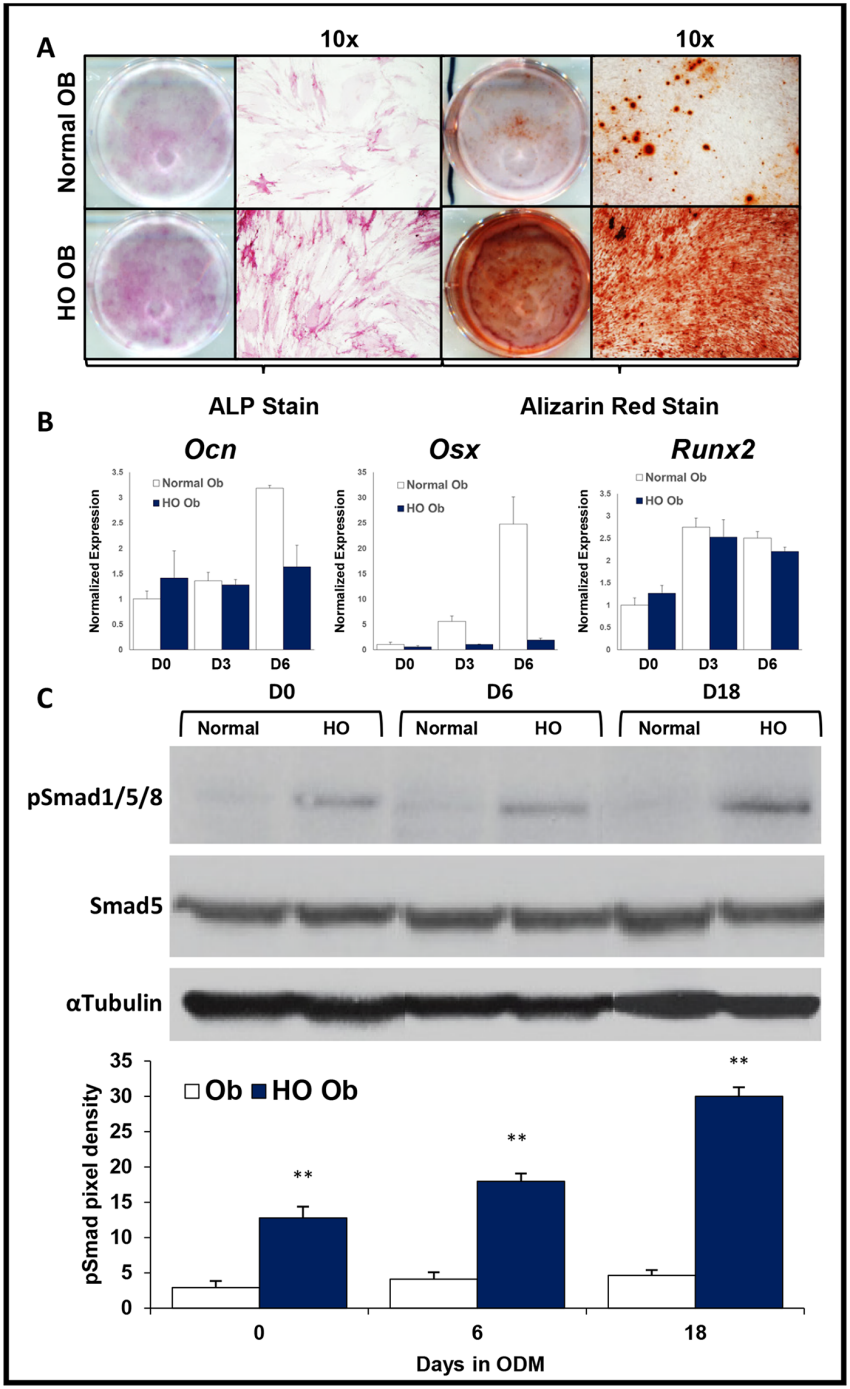
Characterization of *in vitro* osteogenic differentiation of osteoblasts isolated from HO and normal bone. (A) Human osteoblasts were harvested from excised HO (HO-Ob) and surrounding normal bone (Ob). HO derived osteoblasts showed more ALP activity and more in-vitro bone deposition by Alizarin red staining than cells derived from normal bone. Representative wells and 10x micrographs are shown. (B) Gene transcript levels of *Ocn*, *Osx*, and *Runx2*. (C) Western blot analysis of pSmad 5 protein levels in these cells after 0, 6, and 18 days in osteogenic media. Data shown are reported as the mean ± SD of at least three separate experimental samples performed in triplicate. *p<0.05; **p<0.01. Student T-test.

Osteoblasts derived from HO tissue in the transgenic mouse model exhibited increased osteogenic differentiation on the basis of alkaline phosphatase, alizarin red, and gene and protein expression when compared with skeletal osteoblasts (Fig 5). We detected a similar increased mineralization capacity with human HO-derived osteoblasts when compared with skeletal osteoblasts from the same patient (Fig 6). This human finding is surprising given that these cells are likely from more differentiated HO than in our mouse models and that human skeletal osteoblasts demonstrated greater osteogenic transcript activity than their HO counterparts.

## Discussion

Heterotopic ossification (HO) is a debilitating condition which occurs following trauma and in patients with genetic mutations in the type I bone morphogenetic (BMP) receptor *ACVR1*. Studies have attempted to identify the “putative stem/progenitor cells” responsible for HO, however, the numerous cells identified indicates that differing niches likely plays a role in the cell responsible.[37] Therefore, it is possible that the isolation of a single cell type with osteogenic potential may not be entirely representative of the osteogenic niche.

Rather than isolate one lineage of cells based on cell surface markers, we have harvested cells from the tissues of two trauma-induced HO models[16, 17, 20–22], a genetic HO model[24] and from human tissues. In our blast/polytraumatic extremity injury, rats are exposed to a systemic inflammatory insult in the form of a high-energy blast injury followed by trans-femoral amputation. All rats develop heterotopic ossification at the amputation site in this model[20–22]. In the burn/tenotomy model, mice undergo Achilles’ tendon transection followed by 30% total body surface area partial-thickness burn over the dorsum which causes radiographic evidence of HO within 5–6 weeks after injury. At three weeks after injury, we observe histologic evidence of mesenchymal cell proliferation, condensation, and cartilage formation which all suggest a cartilage intermediary of formation[16, 17, 23]. Finally, in the genetic model of HO, the bone morphogenetic protein (BMP) receptor *ACVR1* is mutated to induce constitutive expression (*ACVR1 Q207D*) with expression in cells of the Nfatc1-lineage. These mice develop heterotopic bone lesions along tendon and ligamentous structures from birth[24]. Despite the variety of etiologies and tissues in which HO can develop, our results suggest some important similarities among HO-derived osteoblasts and traumatic soft tissue-derived cells including increased proliferation when cultured *in vitro*, and increased osteogenic differentiation.

In the absence of genetic mutation, human HO-derived osteoblasts exhibit increased proliferation and osteogenic differentiation when compared with normal skeletal osteoblasts. This is similarly true in a transgenic model of HO. This finding suggests that there are intrinsic differences between growing ectopic bone when compared to normal bone, which may be reflective of the aggressive nature of ectopic bone formation. These differences in proliferation may help identify targets for future therapeutic strategies against HO. That HO-derived cells exhibit similar clinicopathologic characteristics further validates their use as a screening tool prior to or in tandem with *in vivo* studies. The finding of increased proliferation and osteogenic differentiation also suggests that these cells are particularly aggressive which is consistent with their locally destructive properties observed clinically.

Furthermore, soft tissue injury portending a high risk of HO development as seen in our burn/tenotomy and blast/polytraumatic extremity injury models yields cells with increased proliferation. Soft tissue-derived cells in these models are derived from two distinct histologic and anatomic locations—in the setting of burn/tenotomy, cells were derived from the Achilles tendon transection site, whereas in the setting of blast/ polytraumatic extremity injury, cells were derived from the primarily muscle components surrounding the amputation stump. Our results demonstrate the cells in both models exhibit higher proliferative capacity than those of the contralateral uninjured hind limb suggests that the trauma site is highly proliferative. This in itself is not surprising; however, we also found that cells from the trauma site of both models were markedly more osteogenic when compared with the contralateral side. The consistency across both models suggests that HO which develops in the setting of tendon injury and muscle injury can be interrogated with similar cellular assays in the *in vitro* setting. These findings are also consistent with those previously reported showing that wounds have osteoproliferative cells with a similar panel to that we have identified (CD34+CD29+CD45-CD31-CD11b-)[35, 36]. Another study found that a similar cell subtype (CD34+CD45-CD31-CD11b-GlyA-) located within muscle has increased osteogenic differentiation capacity *in vitro* in the *absence* of injury^36^. It appears that in both the burn/tenotomy model and the mutant mouse models of HO, an osteoproliferative cell with similar identifying panel exists and may contribute to the overall increased osteogenic differentiation observed *in vitro*.

Our results suggest that when the cells present from HO producing sites are removed from their environment, they continue to exhibit key factors associated with aggressive bone development as observed in HO including proliferation and osteogenic differentiation. Therefore, ectopic bone formation appears to be mediated, in part, by local factors which can be isolated at the trauma site. Previous studies have identified mesenchymal cells from trauma sites that exhibit increased osteogenic differentiation. It is possible that our soft tissue includes this previously defined population and others with osteogenic potential[38–40]. Therefore, the isolated soft tissue may include multiple osteogenic cell populations.

In addition to mesenchymal cells, the trauma site also includes endothelial cells, inflammatory cells such as T-cells, neutrophils and macrophages, and the native tissue (*e*.*g*. myocytes or tenocytes). It is also possible that these cells contribute to osteogenic differentiation either directly, or by eluting cytokines which influence the osteogenic potential of nearby mesenchymal cells. Therefore, use of the aggregate soft tissue may serve as a more representative cell population reflecting the cell types present at the trauma site which develops HO.

Our study has several limitations. First, since we are limited in our human tissues to those patients who have HO resected after mature osteoid has developed. Additionally, though our animal models are well characterized, the inflammation seen in rodents in response to injury is known to differ from that seen in humans[41]. Finally, we realize that the heterogeneous cell populations we initially isolate likely differs from the population that we are studying after passage. By studying cells *in vitro* we are selecting for a group of cells that are plastic adherent. However, the fact that the cells of interest remain more osteogenic *in vitro* indicate that they do mimic what is seen *in vivo* and can be used for analyses. Furthermore, our findings suggest that cells from the trauma site may serve as an inexpensive initial screening tool when evaluating potential therapies in lieu of more expensive *in vivo* studies.

## Supporting Information

S1 Table Gene primers for quantitative RT-PCR. (DOCX)
