# Supplementary material for: Characterization of Cells Isolated from Genetic and Trauma-Induced Heterotopic Ossification
Source: PLoS One. 2016 Aug 5;11(8):e0156253. doi: 10.1371/journal.pone.0156253 (PMC4975503; doi:10.1371/journal.pone.0156253)
Supplement: S1 Table — (DOCX) [file pone.0156253.s001.docx]

|  | Name | Primers (5’-3’) |
| --- | --- | --- |
| Mouse | Ocn | F- CTC ACA GAT GCC AAG CCC A  R- CCA AGG TAG CGC CGG AGT CT |
|  | Opn | F- GCA CTC CAA CTG CCC AAG A  R- TTT TGG AGC CCT GCT TTC TG |
|  | Osx | F- ATG GCG TCC TCT CTG CTT G  R- TGA AAG GTC AGC GTA TGG CTT |
|  | Runx2 | \| F- AGA GTC AGA TTA CAG ATC CCA GG \|  \| \| --- \| --- \|   R- TGG CTC TTC TTA CTG AGA GAG G |
| Rat | Opn | F- TCC AGG AGT TTC CCT GTT TC  R- TGA CCT TGA TAG CCT CAT CG |
|  | Osx | F- CCA ATG ACT ACC CAC CCT TT  R- CAC TAG GCA GGC AGT CAG AA |
|  | Runx2 | F- GCG TCC TAT CAG TTC CCA AT  R- ATC AGC GTC AAC ACC ATC AT |
| Human | Ocn | F- CGG TGC AGA GTC CAG CAA A  R- GGT AGC GCC TGG GTC TCT TC |
|  | Osx | F- GGC TAT GCT AAT GAT TAC CCT CC  R- GTC ATG T CCA GAG AGG TGT AGA |
|  | Runx2 | F- GTA CAG CTT TAA GGA TCC CCT CAA TTC  R- TTG CTA ATG CTT CGT GTT TCC A |

**S1 Table.** Gene primers for quantitative RT-PCR.
